# The association of serum 25-hydroxyvitamin D concentrations with elevated serum ferritin levels in normal weight, overweight and obese Canadians

**DOI:** 10.1371/journal.pone.0213260

**Published:** 2019-03-07

**Authors:** Lalani L. Munasinghe, John P. Ekwaru, Marco F. Mastroeni, Silmara S. B. S. Mastroeni, Paul J. Veugelers

**Affiliations:** 1 School of Public Health, University of Alberta, Edmonton, Alberta, Canada; 2 Post-graduation Program in Health and Environment, University of Joinville Region, Joinville, Brazil; 3 Department of Physical Education, University of Joinville Region, Joinville, Brazil; Holbæk Hospital, DENMARK

## Abstract

In light of the growing body of literature suggesting a beneficial effect of vitamin D on inflammatory response, we hypothesized that vitamin D affects serum ferritin (SF), a biomarker of inflammation. The objective of the present study is to examine the association of serum 25-hydroxyvitamin D [25(OH)D] with elevated SF concentrations indicative of inflammation as no earlier study has done so. Data from 5550 Canadian adults who participated in the 2012/2013 and the 2014/2015 Canadian Health Measures Surveys were analysed. We observed that 9.4% of Canadian adults have elevated SF concentrations and that 35.6% were vitamin D insufficient. Among Canadians with under/normal body weights, those with serum 25(OH)D ≥ 75 nmol/L relative to those with serum 25(OH)D < 50 nmol/L, were substantially less at risk for elevated SF concentrations (OR = 0.24; 95% CI = 0.06, 0.89; p = 0.034). We did not observe this association for overweight and obese Canadians. Canadians of older age, non-white ethnicity, males, those with income above $100,000, those who consumed alcohol, and those with high total cholesterol concentrations and elevated blood pressures were more likely to have elevated SF concentrations. Serum 25(OH)D ≥ 75 nmol/L is likely to provoke anti-inflammatory benefits, but intervention studies that achieve high 25(OH)D concentrations and with long follow up are needed to establish the role of vitamin D on SF.

## Introduction

Serum ferritin (SF) reflects the total body iron storage and is the established nutritional marker of iron status [1–4]. SF is also an acute-phase reactant that elevates in systemic response to inflammation [5–11] and has therefore been identified as a marker of inflammation [6, 12, 13]. In the presence of inflammation, elevated SF may conceal poor iron status [2, 5, 9, 14]. SF has also been identified as a predictor of diabetes [15–18], metabolic syndrome [10, 19] and cardiovascular events [20–22]. The prevention and management of elevated SF is therefore a strategy in the prevention of these conditions [10, 18, 22].

The role of vitamin D in bone and mineral metabolism is well established whereas various other roles have been suggested [23, 24]. With respect to the latter, numerous studies have examined the effect of vitamin D on inflammation [23–27], but little consistency exists across the few studies that examined the association of vitamin D with SF as a marker of inflammation [1, 3, 28–36], and, importantly, none of these studies examined the effect of vitamin D on elevated SF concentrations indicative of inflammation.

Given the anti-inflammatory effect of vitamin D and that SF increases due to inflammation, we hypothesized vitamin D reduces the probability of elevated SF concentrations. Iron deficiency is the most common nutritional deficiency worldwide [37, 38], however, in Canada an estimated 96% of adults have SF concentrations above the reference of 15 μg/L indicating iron sufficiency [39]. Also, vitamin D insufficiency (plasma 25-hydroxyvitamin D [25(OH)D] < 50 nmol/L) is wide spread [40, 41] with an estimated 41% of Canadian adults experiencing vitamin D insufficiency. If our hypothesis is true, a high prevalence of the co-existence of SF sufficiency and vitamin D insufficiency among Canadians may in fact represent a high prevalence of inflammation obscuring iron deficiency. In addition, in light of the fact that SF is a predictor of chronic diseases, confirmation of our hypothesis would pinpoint at SF as a possible mechanistic pathway for the suggested association of vitamin D with diabetes, metabolic syndrome and cardiovascular disease.

## Materials and methods

### Survey, sample design and participants

The Canadian Health Measures Survey (CHMS) collects data from a nationally representative sample of 3–79 years old Canadians living in the 10 provinces across 5 regions: (1) Atlantic (Newfoundland and Labrador, Prince Edward Island, Nova Scotia and New Brunswick), (2) Quebec, (3) Ontario, (4) the Prairies (Alberta, Manitoba and Saskatchewan), and (5) British Columbia [42, 43]. Four percent of the target population, i.e. those living in the three Canadian territories, certain remote regions, on reserves and other Aboriginal settlements in the provinces, full-time members of the Canadian Forces, and the institutionalized population were excluded from the survey [42, 43]. Survey participants were selected using a multi-stage sampling design, and data were collected as an in-home interview to gather demographic, behavioural and in-depth health information followed by the respondent’s visit to a mobile examination centre (MEC) for direct physical measurements and biological specimen collection. The participant’s consent was taken prior to data collection. Additional information on the CHMS and its sampling frame is available on the Statistics Canada website (http://www.statcan.gc.ca) and Data User Guides [42, 43].

For the present study, we analyzed data from the 2012/2013 CHMS (Cycle 3) and 2014/2015 CHMS (Cycle 4). Sixteen sites from each Cycle 3 and Cycle 4 (total of 32 sites) had been randomly selected from 360 eligible collection sites in the 5 regions of Canada using a systematic sampling method with probability proportional to each site’s population size. Within the sites, dwellings were selected randomly and inhabitants within the dwellings were selected through stratified sampling of based on age-groups. To account for seasonality, data were collected periodically throughout windows of 2 years, i.e. January 2012 to December 2013 and January 2014 to December 2015 for Cycle 3 and Cycle 4, respectively.

A total of 6600 adults aged 18 years and above who participated in the CHMS Cycle 3 and Cycle 4 had biological specimens and considered for analysis in the present study. Those who were pregnant, had an insufficient quantity of blood drawn and with incomplete information on SF concentration, serum 25(OH)D concentration and Body Mass Index (BMI) were excluded leaving a sample of 6510. Observations with serum high sensitivity C-reactive protein (hs-CRP) concentrations in excess of 10 mg/L were excluded (n = 620) due to the possibility that such a high concentration was caused by infections and acute inflammations rather than chronic inflammation [44]. Survey participants, who had chemotherapy treatment during past 4 weeks and known to have cancer, kidney disfunction or disease, liver disease or a gall bladder problem and hepatitis were also excluded (n = 340) as these conditions have been shown to affect SF concentrations [13, 14, 45]. Thus, data of 5550 adult participants from Cycle 3 and Cycle 4 were included in the present analysis.

### Laboratory measurements

SF concentrations were measured by two-site sandwich immunoassay on the Siemens ADVIA Centaur XP analyzer (Siemens Healthineers, Erlangen, Germany) with 10.0% of CHMS reference laboratory precision target. The analytical detection limit for SF was 0.5–1650 μg/L. Serum 25(OH)D concentrations were determined using the LIAISON 25-hydroxyvitamin D TOTAL Assay on the DiaSorin Liaison autoimmunoanalyzer (DiaSorin, Ltd, Stillwater, Minnesota) using chemiluminescent immunoassay technology with the analytical detection limit of 10–375 nmol/L. Between-run coefficient of variation for the serum 25(OH)D assay was 13.0% and CHMS reference laboratory precision targets for <20nmol/L, 20–100 nmol/L and >100nmol/L were 15.0%, 10.0% and 12.0%, respectively. Total cholesterol concentrations were analysed using enzymatic reflectance spectrophotometry (CHMS reference laboratory precision target = 3% and analytical detection limit = 1.29–8.40 mmol/L).

### Assessment and measurement of other potential covariates

Gender, age, ethnicity, household income, body weight status, blood pressure status, physical activity level, alcohol consumption, smoking status and sunlight exposure were considered as potential covariates. Age at the time of the study visit was calculated from the date of birth. Household income, a social determinants of health, was available through the survey and categorized as ≤$ 50,000, $ 50,001–100,000 and ≥ $100,001. A fixed stadiometer (Quickmedical 235A, United States) measured the standing height to the nearest 0.01 mm and a digital scale (Mettler Toledo 2256 VLC, United States) measured the body weight to the nearest 0.01 kg at the MEC. They were rounded to 0.01 cm and 0.1 kg, respectively. BMI was calculated as weight/height^2^ (kg/m^2^) and body weight status category was defined using BMI as “underweight”, “normal weight”, “overweight” and “obese”, based on the WHO classification [46]. “Underweight” and “normal weight” categories were combined into a single category due to few underweight adults. The daily activity counts were measured using Respironics Actical Activity Monitors (Oregon, United States), that were distributed to the participants and advised to wear for seven days following their visit. Respondents in wheelchairs were excluded from physical activity measurements. The recorded intensity, timing (day and time), frequency and duration as well as information on sedentary behaviour (excluding sleep) were used to identify the physical activity level as “inactive/low active” or “active/highly active (average steps per day ≥7500)” [47] among those who wore their Activity Monitors for at least 4 days. Others were grouped as “Incomplete data”. Ethnicity was dichotomized as “white” and “non-white” (i.e., Chinese, South Asian, Black, Filipino, Latin American, Southeast Asian, Arab, West Asian, Japanese, Korean, Aboriginal, and other ethnic backgrounds). “Elevated” level of blood pressure was considered as having blood pressure ≥120/80 mmHg based on the most recent guidelines [48] and if participants reported taking medications for high blood pressure. Smoking status included “never smoker”, “ex-smoker” and “current-smoker”. Alcohol consumption was defined as “drinker” and “non-drinker” whereby subjects were considered non-drinkers if their lifetime consumption was less than 100 drinks. The time spent outdoors while at work or home during 10.00 a.m to 4.00 p.m from May to September (i.e. summer months in Canada) was used as a proxy to sunlight exposure and was categorized as “0–≤5 minutes/day”, “>5 minutes/day” and “Not stated/Not applicable”.

### Statistical analyses

CHMS data from both Cycle 3 and Cycle 4 were considered in the present study. Elevated SF level was defined as having serum concentrations of ≥ 200 and ≥ 300 ng/mL for females and males, respectively [49–51]. Serum 25(OH)D concentrations were categorized as “<50”, “50-<75”, and “≥75” nmol/L after LOESS curves had identified two meaningful turning points: one close to 50 nmol/L and one nearing 75 nmol/L, which are commonly used cut offs in vitamin D research. The LOESS curve is depicted in S1 Fig. The association of serum 25(OH)D concentrations with elevated SF concentrations was examined using univariable and multivariable logistic regression models stratified by weight status: under/normal weight, overweight and obesity. The multivariable analyses considered the confounding potential of gender, age, ethnicity, total cholesterol, physical activity level, smoking status, and alcohol consumption. Also household income, season, blood pressure status and sunlight exposure had been considered, but because these variables were not statistically significant, they were not included to preserve statistical power. Hemoglobin concentrations were not considered as a confounder as SF [52], body weight status [4] and serum 25(OH)D [36, 53] affect hemoglobin concentrations. These multivariable analyses were repeated for the subgroups of white and non-white Canadians. However, due to small numbers of non-white Canadians in each of the body weight strata, their multivariate analysis did not provide a good model fit and herewith failed to produce estimates. To characterize the independent importance of the various variable for elevated SF, a multivariable logistic regression model was created with age, gender, total cholesterol, blood pressure status, physical activity level, smoking, alcohol consumption, body weight status, income, ethnicity, region, season and sunlight exposure as independent variables. In order to accommodate the complex sampling design, all analyses were weighted such that the estimates represent national representative estimates for adults in Canada. All analyses were carried out using Stata SE 15.0 (Stata Corp, College Station, TX, USA) and considered a p-value of less than 5% as statistical significant. All processes of CHMS were reviewed and approved by the Health Canada and Public Health Agency of Canada Research Ethics Board. The Health Research Ethics Board of the University of Alberta provided the ethical approval to analyse the CHMS data for the purpose of the present study.

## Results

Participant characteristics (n = 5550) are shown in Table 1. The median SF concentration was 86.0 μg/L (IQR = 39.0, 169.0) and the median serum 25(OH)D concentration was 59.3 nmol/L (IQR = 43.1, 75.2). Of all Canadian adults 9.4% had elevated SF concentrations and 35.6% had insufficient vitamin D levels, i.e. serum 25(OH)D <50 nmol/L [54].

**Table 1:** General characteristics of Canadian adults participating in the 2012/2013 and 2014/2015 Canadian Health Measures Surveys*.

Characteristic	All Canadians	Under/normal weight	Overweight and not obese	Obese
**Serum ferritin, μg/L**				
Median (IQR)	86.0 (39.0, 169.0)	66.0 (32.0, 137.0)	96.0 (48.0, 189.0)	103 (44.0, 194.0)
**Serum ferritin category**^§^**, %**				
Normal	90.6	93.4	88.8	89.4
Elevated	9.4	6.6	11.2	10.6
**Serum 25(OH)D, nmol/L**				
Median (IQR)	59.3 (43.1, 75.2)	60.8 (44.8,78.8)	60.2 (45.0, 75.6)	54.5 (39.8, 70.3)
**Serum 25(OH)D category, %**				
<50 nmol/L	35.6	32.1	34.1	42.8
50–<75 nmol/L	39.2	38.9	39.8	38.5
≥75 nmol/L	25.2	29.0	26.1	18.7
**Age, years**				
Mean (Bootstrap SD)	45.4 (0.2)	41.1 (0.5)	47.7 (0.4)	48.1 (0.6)
**Gender, %**				
Male	52.1	42.3	59.2	55.5
Female	47.9	57.7	40.8	44.5
**Household income, %**				
≤$ 50,000	30.5	31.2	29.6	30.7
$ 50,001–100,000	34.6	35.2	35.5	32.3
≥ $100,001	34.9	33.6	34.9	36.9
**Region of residence, %**				
Atlantic	6.8	5.0	7.6	8.2
Quebec	23.4	24.0	23.3	22.4
Ontario	38.3	36.3	40.2	38.5
Prairies	18.2	18.2	16.4	20.9
British Columbia	13.3	16.5	12.5	10.0
**Ethnicity, %**				
White	78.2	74.0	77.7	85.1
Non-white	21.8	26.0	22.3	14.9
**Body weight status, %**				
Under/normal weight	36.7	-	-	-
Overweight and not obese	37.6			
Obese	25.7			
**Blood pressure status, %**				
Normal	68.1	81.5	62.6	57.0
Elevated (≥ 120/80 mmHg)	31.9	18.5	37.4	43.0
**Serum total cholesterol, mmol/L**				
Mean (Bootstrap SD)	4.8 (0.03)	4.6 (0.05)	4.9 (0.04)	5.0 (0.04)
**Smoking status, %**				
Never-smoker	50.2	56.7	48.5	43.6
Ex-smoker	28.0	20.3	31.0	34.7
Current-smoker	21.8	23.0	20.5	21.6
**Alcohol consumption, %**				
Non-drinker	15.9	16.7	14.7	16.6
Drinker	84.1	83.3	85.3	83.4
**Physical activity level**^†^**, %**				
Inactive/low active	33.3	29.2	34.0	38.3
Active/highly active	40.0	42.4	40.8	35.2
Incomplete data	26.7	28.4	25.2	26.5
**Season, %**				
Spring	18.9	20.2	18.4	17.8
Summer	29.5	28.6	27.5	33.7
Fall	24.3	24.6	26.6	20.5
Winter	27.3	26.6	27.5	28.0
**Sunlight exposure, %**				
0–≤5 minutes/day	21.4	24.1	20.9	18.3
>5 minutes/day	51.6	52.6	50.6	51.6
Not stated/Not applicable	27.0	23.3	28.5	30.1

Abbreviations: 25(OH)D– 25 hydroxyvitamin D; SF–serum ferritin; IQR–inter quartile range

*Results of 5550 participated adults aged ≥18 years were weighted to represent national estimates.

^§^Elevated SF level = SF concentration ≥ 300 ng/mL for males and ≥ 200 ng/mL for females [49–51].

^†^Participants with data entries from Activity Monitors for less than 4 days were considered as “Incomplete data”.

Table 2 depicts the association of serum 25(OH)D concentration with elevated SF adjusted for age, gender, total cholesterol, blood pressure status, physical activity level, smoking, alcohol consumption, and ethnicity, separately for under/normal weight, overweight and obese participants. Serum 25(OH)D ≥75 nmol/L was inversely associated with elevated SF among individuals with under/normal weight (OR = 0.24; 95% CI = 0.06, 0.89; p = 0.034). This association was not statistically significant for those who were overweight or obese.

**Table 2:** Associations of serum 25(OH)D with elevated serum ferritin level and serum ferritin concentrations among under/normal weight, overweight and obese participants of the 2012/2013 and 2014/2015 Canadian Health Measures Surveys*^§^.

Serum 25(OH)D	Under/normal weight		Overweight and not obese		Obesity	
	OR (95% CI)	p value	OR (95% CI)	p value	OR (95% CI)	p value
**Univariable**						
<50 nmol/L	1.00		1.00		1.00	
50–<75 nmol/L	0.40 (0.15, 1.05)	0.062	1.11 (0.57, 2.14)	0.747	0.91 (0.54, 1.53)	0.721
≥75 nmol/L	0.23 (0.09, 0.64)	**0.007**	0.75 (0.46, 1.22)	0.238	0.77 (0.35, 1.72)	0.508
**Multivariable**^‡^						
<50 nmol/L	1.00		1.00		1.00	
50–<75 nmol/L	0.42 (0.14, 1.22)	0.106	1.48 (0.80, 2.72)	0.200	0.89 (0.43, 1.84)	0.745
≥75 nmol/L	0.24 (0.06, 0.89)	**0.034**	1.30 (0.70, 2.26)	0.327	0.69 (0.18, 2.63)	0.574
**White Canadians**^‡^						
<50 nmol/L	1.00		1.00		1.00	
50–<75 nmol/L	0.26 (0.06, 1.13)	0.072	1.44 (0.71, 2.94)	0.293	0.79 (0.36, 1.76)	0.553
≥75 nmol/L	0.17 (0.03, 0.81)	**0.028**	1.53 (0.69, 3.40)	0.278	0.52 (0.13, 2.04)	0.336

Abbreviations: OR, Odds Ratio; CI, Confidence interval

*Results of 5550 participated adults aged ≥18 years were weighted to represent national estimates and adjusted for all covariates in the table.

^§^Elevated SF level = SF concentration ≥ 300 ng/mL for males and ≥ 200 ng/mL for females [49–51].

^‡^ multiple logistic regression models were adjusted for age, gender, total cholesterol, physical activity level, smoking, alcohol consumption, and ethnicity.

Repeating these multivariable analyses while considering of normal weight rather than underweight and normal weight combined revealed estimates for normal weight that closely approximated the estimates for under/normal weight in Table 2. Table 2 further depicts the association of serum 25(OH)D concentration with elevated SF for the ethnic subgroup of white Canadians. The associations in this subgroup of white Canadians were similar to those for Canadians.

Age, gender, income, ethnicity, blood pressure status, serum total cholesterol and alcohol consumption were associated with having an elevated SF level, whereas region of residence, body weight status, smoking status, physical activity level, season and sun light exposure were not associated in a statistically significant manner (Table 3).

**Table 3:** Associations of age, gender, household income, region of residence, ethnicity, body weight status, blood pressure status, total cholesterol, smoking status, alcohol consumption, physical activity level, season and sunlight exposure with elevated serum ferritin level among Canadians participating in the 2012/2013 and 2014/2015 Canadian Health Measures Surveys*.

Covariate	Elevated serum ferritin level^§^	
	OR (95% CI)	p value^‡^
**Age**	1.04 (1.02, 1.05)	**<0.001**
**Gender**		
Male	1.00	
Female	0.26 (0.16, 0.42)	**<0.001**
**Household income**		
≤$ 50,000	1.00	
$ 51,000–100,000	1.35 (0.85, 2.14)	0.195
≥ $101,000	1.77 (1.10, 2.84)	**0.021**
**Region of residence**		
Atlantic	1.00	
Quebec	1.65 (0.64, 4.28)	0.283
Ontario	1.65 (0.69, 3.98)	0.246
Prairies	1.01 (0.33, 3.09)	0.988
British Columbia	1.11 (0.33, 3.70)	0.862
**Ethnicity**		
White	1.00	
Non-white	2.90 (1.53, 5.49)	**0.002**
**Body weight status**		
Under/normal weight	1.00	
Overweight and not obese	1.05 (0.56, 1.95)	0.876
Obese	1.12 (0.63, 1.99)	0.680
**Blood pressure status**		
Normal	1.00	
Elevated	1.80 (1.19, 2.73)	**0.007**
**Serum total cholesterol**	1.26 (1.02, 1.54)	**0.031**
**Smoking status**		
Non-smoker	1.00	
Ex-smoker	1.05 (0.72, 1.54)	0.774
Current smoker	1.47 (0.95, 2.30)	0.080
**Alcohol consumption**		
Non-drinker	1.00	
Drinker	1.63 (1.01, 2.64)	**0.047**
**Physical activity level**^†^		
Inactive/low active	1.00	
Active/highly active	0.86 (0.60, 1.24)	0.403
Incomplete data	1.03 (0.73, 1.46)	0.868
**Season**		
Winter	1.00	
Spring	0.86 (0.30, 2.44)	0.762
Summer	0.83 (0.39, 1.79)	0.626
Fall	0.71 (0.32, 1.58)	0.390
**Sunlight exposure**		
0–<5 minutes/day	1.00	
≥5 minutes/day	1.13 (0.71, 1.78)	0.592
Not stated/Not applicable	0.93 (0.49, 1.77)	0.826

Abbreviations: OR, Odds Ratio; CI, Confidence interval

*Results of 5550 participated adults aged ≥18 years were weighted to represent national estimates and adjusted for all covariates in the table.

^§^Elevated SF level = SF concentration ≥ 300 ng/mL for males and ≥ 200 ng/mL for females [49–51].

^†^Participants with data entries from Activity Monitors for less than 4 days were considered as “Incomplete data”.

## Discussion

We revealed that 9.4% of Canadian adults have elevated SF levels. Under/normal weight adults, compared to overweight and obese adults, had lower SF concentrations and were less likely to experience adverse elevated SF levels. Under/normal weight adults with serum 25(OH)D concentrations ≥75 nmol/L were 0.24 times as likely to have elevated SF compared to under/normal weight adults with serum 25(OH)D concentrations ≤ 50 nmol/L. Serum 25(OH)D concentrations were not associated with elevated SF among overweight and obese individuals. Older age, non-white ethnicity, males, those who had annual income above $100,000, those who consumed alcohol, and those with high total cholesterol concentrations and elevated blood pressures were more likely to have elevated SF concentrations.

Low levels of SF indicate iron deficiency resulting from an inadequate intake of dietary iron or a negative iron balance, often causing so-called ‘iron deficiency anemia’. Elevated levels of SF indicate chronic inflammation, a bodily state whereby the iron absorption is reduced and iron metabolism disturbed, potentially causing ‘anemia of inflammation’ or ‘anemia of chronic disease’. Elevated levels of SF may also conceal the presence of “iron deficiency anemia”. The 2009/2011 CHMS [39], revealed that 97% and 96% of Canadians had sufficient haemoglobin and SF levels, respectively: In other words, the vast majority of Canadians have an adequate iron balance, which is to be expected because Canada, like the US but unlike most other nations, has legislation for mandatory iron fortification of wheat flour [55]. Given the adequate iron supply, the 9.4% of Canadians with elevated SF, observed in the present study, is unlikely concealing any iron deficiency anemia. This is likely different in nations without effective fortification practices.

Of the few studies that examined the associations between vitamin D and SF none had focussed on the effect of vitamin D on reducing the risk of reaching elevated SF concentrations. In addition, none of these studies had been conducted among the general population whilst revealing the effect of vitamin D in population-based samples is particularly important in light of the fact that an elevated SF concentration is both a marker of inflammation and a predictor of chronic disease. For a clinical sample, Sim et al. [36] identified high mean SF levels among vitamin D deficient patients in conjunction with low albumin and low mean hemoglobin concentrations. These high SF levels [56] and low albumin levels [57] reflect the high prevalence of inflammation among vitamin D deficient individuals [58] irrespective of their iron status. Though their primary objective was not to examine the association between SF and vitamin D, some cross-sectional studies did report low SF levels among vitamin D deficient children and adolescents [35] and among patients with inflammatory bowel disease [59]. Contrarily, a recent study [1] demonstrated low SF among vitamin D deficient athletes which was partially explained by their poor nutritional status [60, 61]. None of the above studies had considered potential confounders in the association of vitamin D with SF. A recent analysis of the Korea National Health and Nutrition Survey did consider confounders and reported an inverse association between serum 25(OH)D and SF among Korean men [29]. The authors also reported this association for premenopausal women [29] and for women without metabolic syndrome [28], but not for post-menopausal women [29] and women with metabolic syndrome [28]. The latter, to some extent, seems consistent with the observation of the present study that an association between vitamin D and SF was observed among under/normal weight Canadians, but not among those with overweight and obesity. In terms of intervention studies, in a randomized placebo-controlled trial in healthy adults, 16 weeks of vitamin D supplementation of 10 μg/d (400 IU) or 25 μg/d (1000 IU) did increase serum 25(OH)D concentrations from 28.7 nmol/L to 48.8 nmol/L but did not affect SF concentrations [30]. This could be due to not reaching sufficient serum 25(OH)D concentrations necessary to improve SF levels or could be due to the fact that changes in SF, as a marker of chronic inflammation, is gradual and that a 16 week follow up is therefore too short. We therefore recommend intervention studies that administer higher doses of vitamin D and have longer durations of follow up to establish the effect of vitamin D on SF.

The current vitamin D recommendations assume that 50 nmol/L ensures good bone health [54] whereas the Endocrine Society [62], the National Osteoporosis Society [63], Osteoporosis Canada [64], the Multiple Sclerosis Society of Canada [65], and the American Geriatrics Society [66] recommend higher serum 25(OH)D concentrations (≥75 nmol/L) to ensure a broader spectrum of health benefits. In light of this study’s observations, compliance with the higher recommendations may potentially achieve a lowering of the risk for elevated SF concentrations among under/normal weight subjects. Investigations of another inflammation marker, hs-CRP, had suggested that obese subjects, rather than normal weight subjects, reduce the risk of having elevated hs-CRP serum concentrations by increasing their serum 25(OH)D concentrations [26]. As both elevated SF and elevated hs-CRP concentrations also predict adverse cardiovascular events, higher serum 25(OH)D concentrations may not only reduce inflammation but also cardiovascular disease, though through distinct mechanistic pathways. We recommend intervention studies that achieve high 25(OH)D concentrations and with long follow up to establish these health benefits.

We demonstrated for under/normal weight adults an association between serum 25(OH)D concentrations and the risk of elevated SF, but not for subjects with excess body weight. This may be due to the fact that obesity is a low-grade chronic inflammatory state [67, 68] that itself provokes elevated SF levels [2, 12, 13]. Of note, in addition to obesity, many conventional cardiovascular risk factors such as increasing age [69], male gender [69], high total blood cholesterol [70] and high blood pressure [48, 69–71] were associated with elevated SF in this study. In contrast, the favourable effects of moderate alcohol consumption in lowering cardiovascular risk [72] did not apply to inflammation as we observed a positive association between alcohol consumption and elevated SF. In addition, for physical activity [71] and smoking [70, 71], both established determinants of cardiovascular health, we did not show associations with elevated SF concentrations in the present study.

To our knowledge, this is the first study to demonstrate the association between vitamin D and elevated SF concentrations. A strength of this study is that it made use of the CHMS that has various quality control mechanisms in place and included a large nationally representative sample [42, 43]. While our study found an inverse association between serum 25(OH)D and elevated SF among Canadians, a causal relationship cannot be established due to cross-sectional nature of the CHMS. Whereas we considered various confounders, and more so that other studies did, we may not exclude the role of additional confounding factors such as dietary factors, menopausal status, genetic mutations or unknown causes [14, 29, 45, 73], because this information was not available or because our statistical models could not accommodate more confounders. Caution is therefore warranted when interpreting the results. Further, potential error in the measurement of serum 25(OH)D concentrations with the DiaSorin Liaison Assay [74, 75] should be acknowledged. The present study did exclude subjects with acute inflammation based on elevated hs-CRP concentrations.

## Supporting information

S1 Fig LOESS curve of serum 25(OH)D and serum ferritin concentrations. (PDF)
