# Supplementary figures and images for: The association of serum 25-hydroxyvitamin D concentrations with elevated serum ferritin levels in normal weight, overweight and obese Canadians
Source: PLoS One. 2019 Mar 7;14(3):e0213260. doi: 10.1371/journal.pone.0213260 (PMC6405102; doi:10.1371/journal.pone.0213260)

S1 Fig. LOESS curve of Serum 25(OH)D and Serum Ferritin concentrations

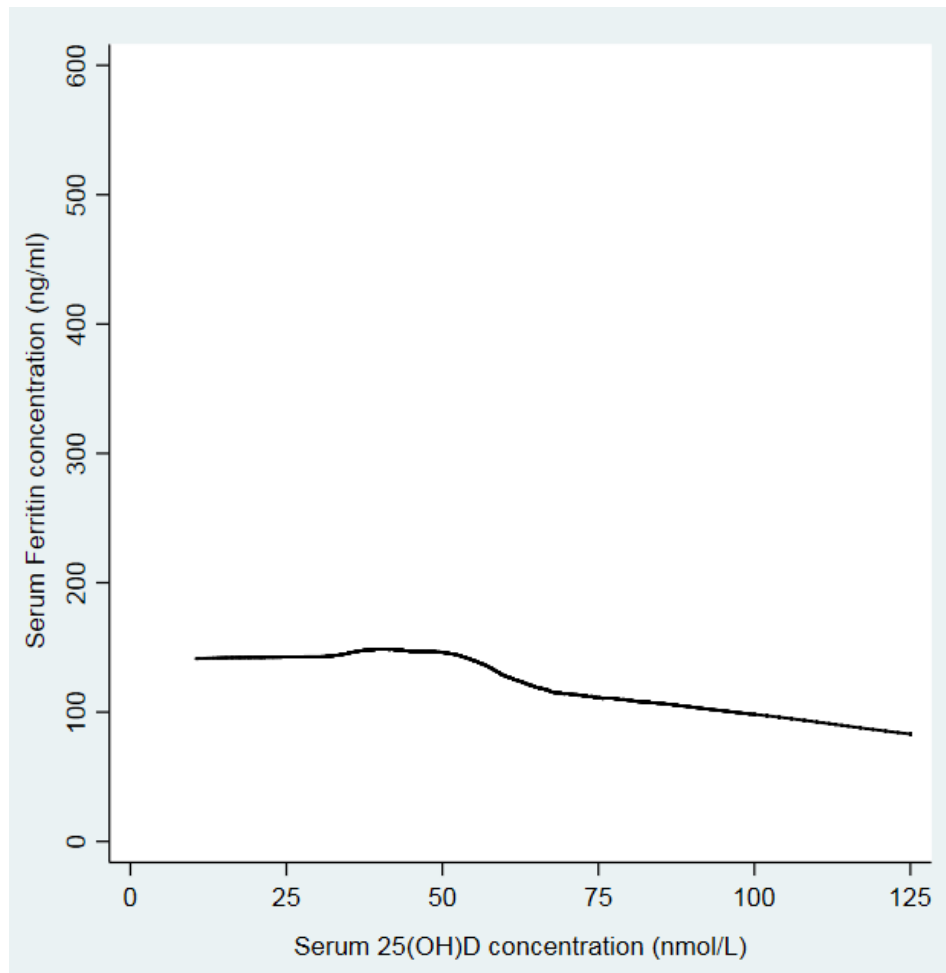

Supplement: S1 Fig — (PDF) [file pone.0213260.s001.pdf]
